# Do Thai Physicians Recommend Seasonal Influenza Vaccines to Pregnant Women? A Cross-Sectional Survey of Physicians’ Perspectives and Practices in Thailand

**DOI:** 10.1371/journal.pone.0169221

**Published:** 2017-01-18

**Authors:** Prabda Praphasiri, Darunee Ditsungneon, Adena Greenbaum, Fatimah S. Dawood, Pornsak Yoocharoen, Deborah M. Stone, Sonja J. Olsen, Kim A. Lindblade, Charung Muangchana

**Affiliations:** 1 Influenza Program, Thailand Ministry of Public Health, U.S. Centers for Disease Control and Prevention Collaboration, Nonthaburi, Thailand; 2 Influenza Division, Centers for Disease Control and Prevention, Atlanta, Georgia, United States of America; 3 Department of Disease Control, Ministry of Public Health, Nonthaburi, Thailand; 4 Centers for Disease Control and Prevention, Atlanta, GA, United States of America; 5 National Vaccine Institute (public organization), Nonthaburi, Thailand; Centers for Disease Control, TAIWAN

## Abstract

**Background:**

Physicians play a major role in influencing acceptance and uptake of vaccines. However, little is known about physicians’ perspectives on influenza vaccination of pregnant women in Thailand, for whom vaccine coverage is estimated at <1%.

**Method:**

In 2013, a self-administered questionnaire on physicians’ perceptions, attitudes and practices related to influenza vaccination for pregnant women was distributed to 1,134 hospitals with an antenatal care clinic (ANC) in Thailand. At each hospital, one physician working at the ANC completed the survey. Predictors of routine recommendation of influenza vaccine were analyzed utilizing log-binomial regression.

**Results:**

A total of 580 (51%) complete responses were received from physicians practicing at ANCs. A favorable attitude towards vaccination was expressed by 436 (75%) physicians, however only 142 (25%) reported routinely recommending influenza vaccine to pregnant women in their current practice. Physicians were more likely to recommend influenza vaccine routinely when they had more than three years of practice (prevalence ratio [PR] 1.9, 95% CI 1.2–2.3), had treated pregnant women for influenza (PR 1.8, 95% CI 1.3–2.7), perceived the influenza vaccine to be effective (moderate level: PR 1.6, 95% CI 1.1–2.4; high level: PR 1.9, 95% CI 1.3–2.9) and were aware of the Ministry of Public Health’s (MOPH) recommendation of influenza vaccination in pregnancy (PR 1.3, 95% CI 1.1–1.7). Vaccine not being available, perception that policy was ambiguous and lack of awareness of MOPH recommendations were the most commonly cited barriers to routine recommendation of influenza vaccine.

**Conclusion:**

Despite a national policy to vaccinate pregnant women for influenza, only 25% of Thai physicians working in ANCs routinely recommend vaccination. Strategies are needed to increase vaccine availability and free vaccine services, address clinician concerns over vaccine effectiveness and expand healthcare provider awareness of MOPH recommendations.

## Introduction

Pregnant women are at an increased risk of complications from influenza [1,2] and some research shows that infants born to mothers with influenza are at increased risk for perinatal outcomes such as pre-term birth and low birth weight [3–6]. Vaccination is regarded as the most effective influenza preventive strategy [7,8] and has been documented to be both safe and effective for preventing influenza in pregnant women [9,10]. In addition, some studies indicate that vaccination of pregnant women may provide protection against influenza to infants during the first few months of life, before they are eligible for vaccination [10–12]. In the past few years, the World Health Organization (WHO) has identified pregnant women as the highest priority group for influenza vaccination, and along with U.S. Centers for Disease Control and Prevention’s Advisory Committee on Immunization Practices (ACIP) [8], recommends vaccination of pregnant women at any time during pregnancy [5].

Vaccination of pregnant women against influenza depends on factors related to both pregnant women and their health care providers [13]. Provider recommendation has been found to be one of the most effective motivators of vaccination among pregnant women [13,14], a phenomenon which is also seen amongst Thai pregnant women [15]. Surveys in the United States have found significant support for influenza vaccination in pregnancy among obstetricians [16, 17] as well as a high rate of routine recommendations for influenza vaccination by providers [18]. However, the rates of vaccination reported by pregnant women are often less than the proportion of physicians who say they support vaccination [16,18–20]. Barriers to influenza vaccination may include lack of awareness of the risks of severe influenza during pregnancy among both physicians and pregnant women, concerns regarding vaccine safety and effectiveness, and difficulty in obtaining influenza vaccine [20–23].

Since 2009, the Thailand Ministry of Public Health (MOPH) has recommended vaccination of all pregnant women during the second and third trimesters [24], and in 2015, pregnant women were considered as the highest priority group [25]. Despite these recommendations, uptake of vaccination among Thai pregnant women has been very poor, with less than 1% of pregnant women in the public health care setting receiving an influenza vaccine between 2010 and 2012 [26]. Although the supply of vaccines has been less than needed [26], little else is known about the barriers to vaccination among pregnant women and the perceptions and practices of physicians in Thailand. In 2013, we conducted a nationwide, cross-sectional survey among physicians in all hospitals providing antenatal care in Thailand to identify providers’ perceptions, attitudes and practices related to influenza vaccination and identify barriers to vaccination of pregnant women.

## Materials and Methods

### Study setting

Thailand is a middle-income country in Southeast Asia with an estimated population of 66.8 million people. The Thai MOPH provides influenza vaccines free of charge to eight high risk groups: elderly ≥65 years of age, children 6–35 months of age, persons with chronic diseases, obese persons, mentally challenged persons, healthcare personnel, poultry cullers and pregnant women. In 2012, Thailand purchased 3.29 million doses of influenza vaccines, out of which 5,394 (0.2%) were administered to pregnant women [26]. Pregnant women were initially considered as a risk group for influenza vaccination in 2009 and elevated to the highest priority group in 2015 [25].

Out of a total of 1,389 hospitals in Thailand, 1,134 (889 public and 245 private) have antenatal clinics that serve around 750,000 pregnant women every year [27]. In Thailand during 2015, there were 2,787 obstetricians/gynecologists among a total 49,910 doctors [28]. Medical doctors must work for three years in a government hospital as a form of internship after graduating from six years of medical training in Thailand.

### Survey instrument

The survey included demographic and clinical practice characteristics along with questions reflecting physician’s perceptions, attitudes, behavior and contextual influences in relation to influenza and influenza vaccination in pregnant women. The questionnaire was refined after a focus group discussion with physicians who attended the 27^th^ annual academic meeting of Royal Thai College of Obstetricians and Gynecologists, Chiang Mai. The main outcome was routine recommendation of influenza vaccine, measured by an affirmative answer to the question, “Do you recommend influenza vaccines to pregnant women in this clinic?” A positive attitude towards influenza vaccination of pregnant women was based on a response of either ‘strongly recommend’ or ‘recommend’ to the question, “What is your opinion towards recommending influenza vaccination to pregnant women?” Perceptions of physicians were considered in terms of disease priority, vaccine safety, vaccine effectiveness and awareness of MOPH recommendations. The questionnaire also included open-ended sections for physicians who did not recommend vaccines to elaborate their reasons and suggest ways to improve the vaccination program.

### Data collection

The survey was conducted between January and April 2013. We mailed the questionnaire, along with a prepaid return envelope, to directors of all public and private hospitals with ANC, according to a government directory of hospitals in Thailand [27]. Hospitals directors were asked to select one Thai physician per ANC to complete the survey, with priority given to obstetricians or gynecologists. One month after the initial mailing, a second round of questionnaires was sent to non-responding hospitals, which were also contacted by telephone. Returned survey forms were checked for completeness, accuracy, and consistency. Respondents with incomplete data were followed up by telephone, where possible. Data were entered into an Access® (Microsoft, Redmond, WA, USA) database and verified by manual comparison with the original questionnaire.

The participants were informed in the questionnaire about the voluntary and confidential nature of participation in the survey. Response to the survey implied their consent. The hospitals were informed, both verbally and in the official letter, that their responses would be used for research purposes. The survey was considered an evaluation of a public health program and exempted from ethical review by the Thai Ministry of Public Health (Nonthaburi, Thailand) and the U.S. Centers for Disease Control and Prevention (Atlanta, GA).

### Data analysis

Data analysis was performed using Stata software version 12.01 (StataCorp LP, College Station, TX, USA). Physicians were categorized by years of practice (≤3 years and >3 years) as those who were still in or had completed their internship. Questions related to perceptions of physicians were recoded into three levels such that higher values represented a greater degree of agreement: 0 = disagree, 1 = uncertain, and 2 = agree. Direction of negatively worded questions was reversed for data analysis. Individual items under the constructs of influenza as a disease priority, influenza vaccine safety, influenza vaccine effectiveness and awareness of MOPH recommendations were averaged to create scores for respective constructs. After case ranking the average scores and examining the nature of its distribution, constructs of influenza as a disease priority and influenza vaccine effectiveness were categorized into tertiles (low/moderate/high), whereas influenza vaccine safety and awareness of MOPH recommendation were categorized into two levels (low/high). Differences in proportions were compared using a Chi square test. Statistical significance was set at P<0.05. Prevalence ratios and 95% confidence intervals were calculated using log binomial regression, in which factors significant in bivariate analysis were adjusted into a final multivariate model using enter method. Responses to open-ended questions were translated into English and grouped into key themes.

## Results

### Response rate

A total of 643 (57%) responses were received from 1,134 hospitals included in the sample; the response rate ranged from 30–70% by province. The responses by public and private hospitals were similar (56% vs 51%, p = 0.31). Among the questionnaires returned, 3 were excluded because of incomplete responses, 21 were discarded because replies were received from non-physicians, and 39 were excluded because they were answered by physicians who did not work in the ANC. The analytic sample thus comprised 580 physicians, out of which 38 (7%) were responses received after the second mailing.

### Demographic and practice characteristics of the respondents

Respondents had a mean age of 38 years (range, 24–77 years), with an average of 13 years in medical practice (range, 1–49 years). A majority of the respondents were male (327; 56%); trained as obstetricians/gynecologists (303; 52%); and had more than 3 years of clinical practice experience (428; 74%) (Table 1). There were 343 (60%) physicians who reported having treated cases of influenza in pregnant women, of whom 29 (8%) physicians had dealt with severe cases. Among the respondents, 124 (21%) reported having received an influenza vaccine in 2009, 239 (41%) in 2010, 322 (56%) in 2011 and 294 (51%) in 2012; overall, 467 (80%) had received an influenza vaccination themselves at least once between 2009 and 2012.

**Table 1:** Characteristics of Thai physician respondents and their perspectives on influenza vaccine (N = 580).

Characteristic	n	%
Age		
<30 years	152	26
30–39 years	204	35
≥40 years	224	39
Sex, male	327	56
Specialty		
Obstetrician/gynecologist	303	52
General practitioner	277	48
Type of hospital		
Public hospital	462	80
Private hospital	118	20
Years in practice		
≤3 years	152	26
>3 years	428	74
Treated case of influenza in a pregnant woman	343	59
Treated case of severe influenza in a pregnant woman (n = 343)	29	8
Received influenza vaccine at least once from 2009–2012	467	80
Have influenza vaccine services in the hospital	543	94
Had opinion of strongly recommending/recommending influenza vaccine to pregnant women	436	75
Ever recommended influenza vaccine for pregnant women	211	36
Routinely recommend influenza vaccine in current practice	142	25

Of the responding hospitals, 543 (94%) reported providing influenza vaccine services. Six percent of public hospitals (28/ 462) and 8% of private hospitals (9/118) reported that their hospital did not provide vaccine. Three quarters of the physicians (436) reported a favorable attitude towards influenza vaccination, 211 (36%) recommended vaccine in the past, and 142 (25%) routinely recommended influenza vaccine to pregnant women in their current practice.

The respondents obtained information regarding safety and effectiveness of influenza vaccine and guidelines mostly through peer discussion (311; 50%) and paper bulletins from the MOPH (308; 49%). Continuing medical education activities (228; 37%) and journal articles (223; 36%) were also identified as sources of information about vaccines for the physicians.

### Physician perceptions

Most respondents agreed that influenza was not limited to mild illness (467; 81%), caused significant illness among the general population (415; 72%), and should be considered a high priority illness (413; 71%) (Table 2). Only one-fifth of the physicians (113; 20%) believed that influenza caused a great deal of illness among pregnant women but most (496; 85%) understood that pregnant women were at increased risk of developing severe influenza. Most physicians considered influenza vaccine safe for a pregnant woman (398; 69%) and fetus (373; 65%). A majority of physicians believed that influenza vaccine was effective at preventing pregnant women from getting sick from influenza (353; 61%) but fewer agreed that vaccinating the mother protected infants during their first six months of life (222; 38%).

**Table 2:** Key perceptions of Thai physicians related to influenza vaccination (N = 580).

Factor	Agree	Disagree	Don’t know
	n (%)	n (%)	n (%)
**Disease Priority**			
Influenza causes substantial disease burden	467 (81)	88 (15)	25 (4)
Influenza causes a great deal of illness among the general population	415 (72)	132 (23)	33 (5)
Influenza is a high priority illness	413 (71)	124 (22)	43(7)
Influenza causes a great deal of illness among pregnant women	113 (20)	379 (65)	88 (15)
Pregnant women are at increased risk for developing severe influenza	496 (85)	50 (9)	34 (6)
**Safety of influenza vaccine**			
Influenza vaccine is safe for pregnant women	398 (69)	29 (5)	153 (26)
Influenza vaccination of pregnant women is safe for their fetus	373 (65)	24 (4)	183 (31)
**Effectiveness of influenza vaccine**			
Influenza vaccine is an effective way to prevent pregnant from getting sick from influenza	353 (61)	52 (9)	175 (30)
Vaccinating pregnant women protects infants during the first six months of life	222 (38)	60 (10)	298 (52)
**Awareness of MOPH**^1^ **recommendations**			
MOPH advises influenza vaccine for pregnant women	431 (74)	46 (8)	103 (18)
MOPH prioritizes pregnant women for receiving influenza vaccine	374 (65)	77 (13)	129 (22)
MOPH recommendations regarding influenza vaccination of pregnant women are clear	81 (14)	363 (63)	136 (23)

^1^MOPH: Ministry of Public Health.

Most physicians knew that MOPH recommended influenza vaccine for pregnant women (431; 74%) while a smaller number understood that pregnant women were one of the prioritized groups for vaccination (374; 65%). Almost two thirds of physicians (363; 63%) deemed the MOPH recommendations related to influenza vaccination of pregnant women as unclear. About 40% (232) of the respondents assumed that recommending influenza vaccine to pregnant women was primarily the responsibility of obstetricians/gynecologists, while 159 (27%) believed the responsibility belonged to nurses and 112 (19%) thought it belonged to general practitioners.

### Bi-variate and multivariate analysis

To compare perceptions about influenza vaccine between physicians who did and did not recommend the vaccine to pregnant patients, we analyzed “agree responses” for individual perceptions using only physicians who worked at hospitals that had influenza vaccine services (n = 543). In comparison to physicians who did not recommend influenza vaccine to pregnant women, significantly higher proportion of vaccine recommending physicians recognized the increased risk of severe influenza in pregnant women, perceived influenza vaccine to be safe for pregnant women and their fetus, considered influenza vaccination of pregnant women to be effective for preventing influenza infection in pregnant women and their offspring till six months of age, and were aware of MOPH policy recommendations (Table 3).

**Table 3:** Comparison of key perceptions among Thai physicians who recommend influenza vaccine and those who don’t in hospitals with influenza vaccine services (N = 543).

Factor	Recommending vaccine		p-value
	Yes (N = 142)	No (N = 401)	
	n (%)	n (%)	
**Disease Priority**			
Influenza causes substantial disease burden	118 (83)	321 (80)	0.73
Influenza causes a great deal of illness among the general population	104 (73)	289 (72)	0.98
Influenza is a high priority illness	108 (76)	285 (71)	0.56
Influenza causes a great deal of illness among pregnant women	31 (22)	76 (19)	0.58
Pregnant women are at increased risk for developing severe influenza	131 (92)	329 (82)	0.003*
**Safety of influenza vaccine**			
Influenza vaccine is safe for pregnant women	115 (81)	257 (64)	0.001*
Influenza vaccination of pregnant women is safe for their fetus	114 (80)	237 (59)	<0.001*
**Effectiveness of influenza vaccine**			
Influenza vaccine is an effective way to prevent pregnant from getting sick from influenza	109 (77)	225 (56)	<0.001*
Vaccinating pregnant women protects infants during the first six months of life	72 (51)	136 (34)	<0.001*
**Awareness of MOPH**^1^ **recommendations**			
MOPH advises influenza vaccine for pregnant women	130 (91)	281 (70)	<0.001*
MOPH prioritizes pregnant women for receiving influenza vaccine	120 (84)	241 (60)	<0.001*
MOPH recommendations regarding influenza vaccination of pregnant women are clear	37 (26)	88 (22)	0.005*

^1^MOPH: Ministry of Public Health.

*Statistically significant at p-value<0.05, p-value obtained from chi square tests.

In an unadjusted model, factors associated with increased physician recommendation of influenza vaccine to pregnant women were training as an obstetrician/gynecologists (PR 1.6; 95% CI 1.2–2.2), having more than three years of clinical experience (PR 2.3; 95% CI 1.4–3.6), having treated pregnant women with influenza in the past (PR 2.4; 95% CI 1.6–3.4), having received influenza vaccine themselves (PR 1.7; 95% CI 1.1–2.7), high perceived vaccine safety (PR 2.1; 95% CI 1.4–3.0), moderate or high perceived vaccine effectiveness (moderate level: PR 2.1, 95% CI 1.4–3.2; high level: 2.6, 95% CI 1.8–3.9) and high awareness of the MOPH’s recommendations (PR 1.5, 95% CI 1.1–2.0) (Table 4).

**Table 4:** Associated factors for vaccine recommendation among respondent physicians (N = 543).

Factor	Recommend influenza vaccine		Crude PR	95% CI	Adjusted PR	95% CI
	Yes (N = 142)	No (N = 401)				
	n (%)	n (%)				
Sex: Female	53 (37)	181 (45)	1			
Male	89 (63)	220 (55)	1.2	0.9–1.7		
Specialty:						
General practitioner	49 (35)	204 (51)	1		1	
OB\GYN	93 (65)	197 (49)	1.6	1.2–2.2*	0.9	0.7–1.3
Type of hospital						
Private hospital	38 (27)	77 (19)	1			
Public hospital	104 (73)	324 (81)	0.7	0.5–1.0		
Years in practice						
≤3 years	18 (13)	120 (30)	1		1	
>3 years	124 (87)	281 (70)	2.3	1.4–3.6*	1.9	1.2–3.2*
Treated pregnant women suffering from influenza	111 (79)	215 (54)	2.4	1.6–3.4*	1.8	1.3–2.7*
Received influenza vaccine themselves in 2009–2012	125 (88)	314 (78)	1.7	1.1–2.7*	1.4	0.9–2.3
Disease Priority						
Low (reference)	51 (36)	170 (43)	1			
Moderate	55 (39)	145 (36)	1.1	0.8–1.6		
High	36 (25)	86 (21)	1.2	0.8–1.6		
Safety of influenza vaccine						
Low (reference)	31 (22)	172 (43)	1		1	
High	111 (78)	229 (57)	2.1	1.4–3.0*	1.3	0.9–2.0
Effectiveness of influenza vaccine						
Low (reference)	29 (20)	178 (44)	1		1	
Moderate	52 (37)	122 (31)	2.1	1.4–3.2*	1.6	1.1–2.4*
High	61 (43)	101 (25)	2.6	1.8–3.9*	1.9	1.3–2.9*
Awareness of MOPH recommendation						
Low (reference)	53 (38)	206 (52)	1		1	
High	88 (62)	190 (48)	1.5	1.1–2.0*	1.3	1.1–1.7*

Abbreviations: PR, Prevalence ratio; CI, confidence interval; OB/GYN, obstetrician/gynecologist; MOPH, Ministry of public health.

*Statistically significant at p-value<0.05, PR and CI calculated by log-binomial regression

In the adjusted model, only four factors remained significantly associated with recommending vaccine: more than three years of clinical experience (PR 1.9, 95% CI 1.2–2.3), prior treatment of influenza cases in pregnant women (PR 1.8, 95% CI 1.3–2.7), moderate to high levels of perception of influenza vaccine effectiveness (moderate level: PR 1.6, 95% CI 1.1–2.5; high level: 1.9, 95% CI 1.3–2.9) and high awareness of MOPH’s recommendations (PR 1.3, 95% CI 1.1–1.7) (Table 4).

### Perceived barriers

Physicians who did not recommend influenza vaccine (401) cited lack of awareness of MOPH recommendations for pregnant women (281; 70%), refusal of vaccine by pregnant women (253; 63%), lack of vaccines in their facility (241; 60%) and cost of vaccines not being covered by insurance (243; 60%) as important barriers to vaccination of pregnant women. Compared to private doctors, physicians working in public hospitals were more likely to identify organizational barriers such as unavailability of vaccine, challenges related to vaccination services in the clinic/hospital or logistics involved in procurement and administration, and inadequate staff and storage facilities (Table 5).

**Table 5:** Comparison of reasons for not recommending vaccines to pregnant women by Thai physicians in public and private hospitals (N = 401).

Reasons	All (N = 401)	Public (N = 326)	Private (N = 75)	p-value
	n (%)	n (%)	n (%)	
I am not aware of recommendations suggesting influenza vaccination of pregnant women	281 (70)	222 (68)	59 (78)	0.07
Pregnant women refuse influenza vaccine	253 (63)	206 (63)	47 (63)	0.93
Not enough influenza vaccine available in the facility	241 (60)	215 (66)	26 (35)	0.001*
The cost of the vaccines are not covered by any health insurances	243 (60)	201 (62)	42 (56)	0.36
This clinic does not provide influenza vaccine	205 (51)	178 (55)	27 (36)	0.04*
Influenza vaccine is not safe for pregnant women	196 (49)	164 (50)	32 (43)	0.23
Pregnant women do not need any vaccines	183 (46)	145 (44)	38 (51)	0.33
Influenza vaccination of pregnant women is not safe for a fetus	196 (49)	164 (50)	32 (43)	0.23
Other logistics involving in procuring or administering influenza vaccine	140 (35)	126 (38)	14 (19)	0.001*
There are not adequate storage facilities to keep vaccine	138 (34)	124 (38)	14 (19)	0.001*
There are not adequate staff to administer vaccine	132 (33)	117 (36)	15 (20)	0.008*

* Statistically significant at p-value<0.05, p-value obtained from chi square tests.

Of 211 physicians who had recommended influenza vaccine in the past, 69 (31%) discontinued the practice of recommending vaccines. We grouped their open ended reasons for not recommending vaccine into key themes, which are presented in Table 6.

**Table 6:** Open-ended responses of Thai physician to perceived barriers of influenza vaccination of pregnant women.

Discontinued the practice of recommending vaccines (N = 69)	n (%)
**Key themes of reported reasons:**	
a) Gaps in policy implementation^1^	30 (43)
b) Unavailability of vaccine and services in the practice	28 (41)
c) Perceived benefit of vaccine only in outbreaks	25 (36)
d) Perceived risk of side effects and complications of vaccine	20 (29)
e) Limited provision of vaccines^2^	15 (22)

^1^Responses such as policy and strategy unclear, no vaccine campaigns, confusion about the vaccines being free or not

^2^Vaccines provided by MOPH are limited to the vaccine campaign which lasts only for a few months in a year

### Suggestions from physicians

The physicians suggested more training programs to update their understanding of the vaccine; and adding the vaccine in patient record book to incorporate influenza vaccine as a part of routine ANC services.

## Discussion

We surveyed over 600 physicians providing care for pregnant women and found that only one in four physicians actually recommended vaccine to pregnant women although most Thai physicians have a positive attitude towards influenza vaccination. Physicians were more likely to recommend influenza vaccine routinely when they had more than three years of practice, had treated pregnant women for influenza, perceived influenza vaccine to be effective and were aware of Ministry of Public Health’s (MOPH) recommendation of influenza vaccination in pregnancy. Understanding factors associated with Thai physicians’ decisions to recommend influenza vaccine to pregnant women is critical because physician recommendation of influenza vaccine is a key factor in Thai pregnant women’s decision to get vaccinated during pregnancy [15].

In our study, organizational barriers were frequently cited by physicians who did not recommend influenza vaccine to pregnant women. These barriers include challenges with vaccine availability, storage, and reimbursement, consistent with organizational barriers identified in some other settings [16,22,29,30]. Although the Thai MOPH recommends vaccine for pregnant women, we found that some hospitals do not provide that service. Many physicians were unaware of MOPH recommendations for influenza vaccination and that vaccine is available free of charge to pregnant women. Physicians working in public hospitals were more likely to cite these barriers than private physicians which suggests gaps in policy implementation. Similar problems also led some physicians in our study to discontinue recommending influenza vaccines altogether.

Disagreement among healthcare providers about who is responsible for discussing and recommending influenza vaccination is another important barrier to vaccination of pregnant women. We found that physicians were divided in their opinions about who was primarily responsible for recommending influenza vaccine to pregnant women with 27% of providers believing the primary responsibility belonged to nurses and 19% to general practitioners. These findings are consistent with another study from Canada that found that obstetricians were more likely to support vaccination of pregnant women compared to family physicians, but less likely to offer it because they believed it was the responsibility of family physicians or local public health units to recommend vaccination [30].

In our study, nearly one third of physicians did not believe influenza vaccine was safe for pregnant women and/or their developing fetuses or effective at preventing influenza in pregnant women. Perception that vaccine is effective was associated with recommending the vaccine to pregnant women. Physicians’ perceptions that vaccines are not safe or effective have also been identified in other studies as key factors associated with not recommending or providing vaccine to pregnant women and thus represent an area for targeted intervention [17,18]. In our study, MOPH bulletins and peer discussion were the most common sources of information about vaccine used by physicians. Therefore, strategies for improving physician knowledge about influenza vaccines could include MOPH bulletins and use of continuing medical education programs or other hospital seminars to promote peer discussion. Educational outreach could also include case studies of the clinical presentation of influenza in pregnant women to improve awareness of influenza among physicians who have not seen pregnant women with mild or severe influenza in their practices. In addition, programs targeting recently graduated doctors may be warranted as our findings indicate that less experienced physicians were less likely to recommend influenza vaccine.

Our study also converged with previous research in finding that providers who themselves were vaccinated were more likely to recommend influenza vaccine to pregnant patients [20]. Physician vaccination could serve as a cue to action, such that MOPH efforts to increase vaccination of healthcare workers may have a secondary benefit of increasing vaccination of pregnant women. Some physicians in our study also suggested adding influenza vaccine to the list of services offered at ANC clinics as making influenza vaccine a part of routine ANC service may increase physicians’ practice of recommending the vaccine.

Our study provides much-needed information about clinician knowledge, attitudes, and practices related to influenza vaccination in Thailand where recommendations for influenza vaccination among pregnant women are relatively new. Our sample includes representation from all Thailand provinces and provides a snapshot of the perspectives of Thai physicians. However, our study also has several limitations. Our sample may not be representative of all physicians working in ANCs as only one clinician was selected from each hospital and respondents were chosen by hospital directors. This targeted sampling was done to elicit responses from key persons responsible for ANC service in Thailand but may have biased results in favor of those physicians most likely to recommend influenza vaccination or have a favorable attitude towards vaccination. In addition, the survey was conducted in 2013 when pregnant women were recommended for influenza vaccination but not yet identified as the highest priority group for vaccination by Thai MOPH.

In conclusion, our study highlights the situation faced by physicians in their local context along with information to formulate strategies aimed at improving influenza vaccination prescription by physicians and acceptance by pregnant women. Some of the key messages that have emerged from this study include the need to increase vaccine availability and free vaccine services in ANCs in Thailand; address physicians’ concerns over vaccine effectiveness and safety; increase influenza vaccination coverage among physicians; expand healthcare provider awareness of MOPH recommendations; and target additional outreach to physicians providing care to pregnant women, especially those with less experience.

## Supporting Information

S1 Codebook For Dataset (XLSX)

S1 Dataset (CSV)

## Abbreviations

ANC: Ante-natal care clinic
MOPH: Ministry of Public Health
WHO: World Health Organization
ACIP: Advisory Committee on Immunization Practices
